# Gout as a Risk Factor for Age-Related Macular Degeneration in Taiwanese Adults—A Population-Based Study in Taiwan

**DOI:** 10.3390/ijerph191610142

**Published:** 2022-08-16

**Authors:** Min-Huei Hsu, Chia-An Hsu, Shih-Chung Lai, Ju-Chuan Yen

**Affiliations:** 1 Graduate Institute of Data Science, College of Management, Taipei Medical University, Taipei 11042, Taiwan; 2 Department of Neurosurgery, Shuang Ho Hospital, Taipei Medical University, Taipei 23561, Taiwan; 3 Department of Ophthalmology, Taipei Veterans General Hospital, Taipei 11217, Taiwan; 4 Department of Ophthalmology, Shuang Ho Hospital, Taipei Medical University, Taipei 23561, Taiwan; 5 Graduate Institute of Biomedical Informatics, College of Medical Science and Technology, Taipei Medical University, Taipei 11042, Taiwan; 6 Department of Ophthalmology, Ren-Ai Branch, Taipei City Hospital, Taipei 10341, Taiwan; 7 Department of Education and Research, Taipei City Hospital, Taipei 10341, Taiwan; 8 University of Taipei, Taipei 10048, Taiwan

**Keywords:** age-related macular degeneration, gout, Taiwan National Health Insurance Research Database

## Abstract

The relationship between gout and age-related macular degeneration (AMD) was suggested in previous literature but has yet to be accepted fully among physicians. This study aimed to explore the effect of gout on the development of age-related macular degeneration in Taiwan. A retrospective cohort study was conducted using Taiwan’s National Health Insurance Database that includes a 2-million-persons dataset. The crude hazard ratio, Kaplan–Meier plot, and separate cox proportional hazard ratio were utilized to demonstrate the effect of gout on the development of age-related macular degeneration. The crude hazard ratio for gout patients developing AMD was 1.55 and the adjusted hazard ratio 1.20. In conclusion, gout is a risk factor for developing AMD, and achieving good disease management is therefore essential for preventing AMD from occurring.

## 1. Introduction

Age-related macular degeneration (AMD) is a vision-threatening illness that affects patients predominantly from the age of sixty onwards. AMD causes reduced central vision, which has a significant impact on patient quality of life and can lead to an increased risk of falls and/or fractures [1]. The disease burden of AMD is therefore substantial, particularly as populations become increasingly aged. The number of patients with AMD is predicted to reach 288 million by 2040 [2]. Unfortunately, the etiology of AMD remains unknown, but risk factors include an age greater than 60, smoking, Caucasian ethnicity, obesity, the presence of comorbidities (such as cardiovascular or other systemic diseases), and a family history of or genetic predisposition to AMD [3]. AMD is clinically classified as early or late stage based on drusen size, drusen numbers, retinal pigmentation around the macula and non-macular retina, neovascularization, and outer retinal thinning. The late stage of AMD is categorized as exudative, wet, neovascular, dry, atrophic, or geographical. In the past two decades, inflammation has been identified as a component in the disease process, particularly involving complement pathway dysregulation. It has been suggested that an imbalanced control of the complement, lipid, angiogenesis, and extracellular matrix results in inflammatory processes, which leads to AMD. Studies have identified 52 common variants at 34 genetic loci that were found to be independently associated with AMD [2]. The complement pathway, which includes CFH (Complement Factor H), CFI (Complement Factor I), C2 (Complement Component 2), CFB (Complement Component Factor B), and C3 (Complement Component 3), accounts for a large proportion of these loci, as well as (to a lesser extent) the age-related maculopathy susceptibility (ARMS2) locus, which acts through a matrix metalloproteinase inhibitor. The association between AMD and inflammation has been determined based on this hypothesis, particularly when it occurs with a background of cardiovascular disease, chronic hepatitis C, or dementia [3]. No cure has yet been found for AMD, and no treatment is available for the geographic or atrophic subtypes. Intravitreal injection of anti-vascular endothelial growth factor (VEGF), such as ranibizumab, aflibercept, and off-label use of bevacizumab, have been used to slow disease progression to vision loss in neovascular or exudative subtypes. To preserve better vision and quality of life in the elderly population, it is therefore necessary to identify risk factors for AMD and prevent further development of the disease [4].

Gout is a chronic metabolic disease that typically presents with hyperuricemia and monosodium urate (MSU) crystal depositions in joints. The presence of these crystals can lead to inflammation, which is known as a gout flare [5], and some individuals become chronic cases who suffer intermittent flare and remission cycles. The prevalence of gout has been reported to be from 0.68% to 3.90% for adults in Asia and Europe [6], but this could be higher in Oceania, where it has been reported to be as high as 8.5% in Maori populations and 13.9% in the Pacific peoples of New Zealand [7]. Furthermore, gout predominantly affects the male gender, and the male to female ratio ranges from 2:1 to 8:1, depending on the ethnicity. Some reports have shown that this difference in prevalence between genders could decrease after the menopause in females. Hyperuricemia can result from the poor degradation of purines or under-excretion of urate, or both. However, not all individuals with hyperuricemia will go on to develop gout, as half of those with hyperuricemia of 10 mg/dl could remain gout-free for up to 15 years. This suggests that there must be an interaction between genetic (including epigenetic) and environmental factors that cause gout to occur in some hyperuricemic patients. Hyperuricemia remains the strongest risk factor for developing gout. Gout flares result from the deposition of MSU crystals in the joints of vulnerable individuals, particularly if they are in a cold environment, have a physiological pH of 7 to 10, or have a higher concentration of serum sodium. MSU crystals tend to be deposited in the lower limbs first, particularly in the first metatarsophalangeal joint of the foot. The deposition of MSU crystals recruits monocytes or macrophages to the site, which induces an innate immune response that results in joint damage [8]. NOD-, LRR-, and pyrin domain-containing protein 3 (NLRP3) is one of the key players in this response. It encodes the NOD-, LRR-, and pyrin domain-containing protein [3], which causes inflammation through a two-signal initiation system [9]. The first signal results in the galvanization of NF-κB through TLR4 and TLR2, with the formation of pro-IL-1β [10] and inflammasome elements. The second signal is derived from MSU crystals, which further promote the recruitment of the inflammasome and trigger caspase-1, which proteolyzes pro-IL-1β to mature IL-1β. IL-1β then interacts with the IL-1β receptor, leading to a cascade of downstream cytokine and chemokine storms. This results in the attraction of neutrophils to the site of MSU crystal deposition, which promotes either a higher level of inflammation or resolution of the gout flare through extracellular traps [11].

Recent research published by Singh et al. [12] noted an association between gout and AMD in elderly patients in the United States, based on the Medicare claims data. They found a hazard ratio (HZ) of a range from 1.25 to 1.39, compared to the matched non-gout elderly, to develop value AMD with different adjustment models. The association between gout and ocular diseases has been investigated by researchers since the beginning of this century. A study by Klein et al. found that the development of pure geographic atrophy in patients with gout had a hazard ratio of 3.48, with a 95% confidence interval of 1.27–9.53 [6]. A case report by Jiang et al. [13] delineated the retinal parafoveal involvements of refractile crystalline retinopathy in a patient with preceding chronic gout. If gout is associated with a higher risk of AMD, gout patients should be notified and treated aggressively early in their clinical course to prevent the future development of AMD and a detrimental impact on quality of life.

The Taiwanese government launched mandatory National Health Insurance (NHI) with universal coverage on 1 March 1995 [14]. Since then, the coverage rate has been over 99%, and as of May 2022, NHI covered 24 million people in Taiwan. In addition, the Bureau of NHI has released claims data on the National Health Insurance Research Database (NHIRD) since 1997, which includes outpatient visits, emergent care, and inpatient services reimbursement data. To prevent future cases of AMD, we sought to determine whether gout is a risk factor for later AMD development and to compare the relative hazard ratios between gout patients and their cohorts using a longitudinal case–control retrospective observational cohort study, with data.

## 2. Materials and Methods

### 2.1. Database

The NHI in Taiwan has released data on the NHIRD since 1997. The information in this database is anonymized prior to release. The NHIRD includes data on claims, such as codes for diagnosis, medication, and intervention, as well as the expenditures for outpatient, emergent, and inpatient care. We therefore utilized this dataset to investigate the association between gout and AMD.

The study was approved by the institutional review board of Taipei Medical University (TMU-JIRB N201602088), which complies with the Declaration of Helsinki. Informed consent was waived because the study used retrospective administrative claims data.

### 2.2. Identification of Cases and Controls

For the case group, this study used data from 16,545 patients aged over 50 years and less than 90 years, who were given a diagnosis of gout (code ICD-9-CM 274) from 1 January 2006 to 31 December 2012. Data from 82,725 patients who did not have a diagnosis of gout were included as a control group, after matching the date, age, sex. The matching ratio of case to control cohort was one to five. To be included in the case group, patients had to have at least three visits recorded with gout ICD-9-CM diagnosis codes. We defined the index date as the date of the first use of gout ICD-9-CM codes.

### 2.3. Exposure Assessment

In addition, we also traced further outpatient visits, emergent care, or inpatient services from the index date for both the case and control cohort groups. The inclusion criterion of exposure was if there had ever been two diagnoses in the outpatient care or one diagnosis in inpatient services of AMD ICD-9 CM codes after the index date. The AMD ICD-9 CM codes were: 365.20 unspecific macular degeneration, 365.21 nonexudative senile macular degeneration of retina, 365.22 exudative senile macular degeneration of retina, and 365.27 drusen, degeneration of macula. To accurately assess exposure, cases of AMD were only included if they occurred more than 180 days following the initial diagnosis of gout.

### 2.4. Statistical Analysis

Statistical Analysis System (SAS) for Windows 9.4 (SAS Institute, Inc., Cary, NC, USA) was used for statistical analysis in this study. Descriptive statistical analyses were carried out to compare the features of the cohorts in terms of demographics and risk of developing AMD.

The risk of AMD in gout patients and controls was compared by estimating the crude hazard ratio with logistical regression. Logistical regression is used widely in the analysis of categorical data, particularly for data with binary variables. It can predict a dichotomous outcome using independent variables; in this study, the dichotomous outcome was the presence or absence of AMD. Kaplan–Meier analysis was used to calculate the cumulative incidence rates of developing AMD between the cohorts, and the log-rank test was used to analyze the differences between the survival curves.

Separate Cox proportional hazard regressions were then performed to determine the AMD-free rate, after adjusting for possible confounding factors (such as age and sex). Cox regression is a method of investigating the effect variables may have on the time taken for a specified event to occur. The crude hazard ratio was calculated after matching and was estimated by univariable Cox regression stratified on matched sets with robust variance estimator to account for the within-matching-set correlation, and the adjusted hazard ratio was calculated after matching and was estimated by multivariable Cox regression stratified on matched sets with robust variance estimator to account for the within-matching-set correlation [15]. The coefficients in Cox regression relate to hazard; a positive coefficient indicates a worse prognosis, and a negative coefficient indicates a protective effect for the variable with which it is associated. Statistical significance taken as a *p*-value of ≤0.05. Comorbidities used in the model included the diagnoses as follows: hypertension (ICD-9-CM 401–405); coronary artery disease (ICD-9-CM: 410–414); diabetes mellitus (ICD-9-CM: 250-); acute and subacute iridocyclitis (ICD-9-CM 364.0x); chronic iridocyclitis (ICD-9CM 364.1x); certain type of iridocyclitis (ICD-9CM 364.2x); unspecified iridocyclitis (ICD-9-CM 364.3x); liver cirrhosis (ICD-9-CM 571); glaucoma (ICD-9-CM 365); hyperlipidemia (ICD-9-CM 272); obesity (ICD-9-CM 278); migraine (ICD-9-CM 346) [16].

## 3. Results

From this dataset, between the period of 1 January 2006 and 31 December 2012, 16,545 gout patients were included in the case group and 82,725 patients in the control group. The mean ages in the case and control groups were 65.44 and 65.43 years, respectively, and were not found to be significantly different. The sex ratio (male to female) for the case and control groups was approximately 2:1 (66.65:33.35) for both. The age group ratio of the case and control cohorts was as follows: 18.915%:18.97% in the age group of 50 to 55 years, 18.84%:18.74% in the age group of 55 to 60 years, 13.65%:13.76% in the age group of 60 to 65 years, 14.45%:14.36% in the age group of 65 to 70 years, 12.85%:12.94% in the age group of 70 to 75 years, and 21.30%:21.25% in the group aged ≥75 years. The compositions of the age groups were very similar between the case and control cohorts and were not found to differ significantly. The median follow-up years and interquartile ranges for the case and control cohorts were 4.15 years (2.38, 5.65) and 4.16 years (2.4, 5.65), respectively. These were not found to be significantly different (*p* = 0.4232) (Table 1).

**Figure 1:**
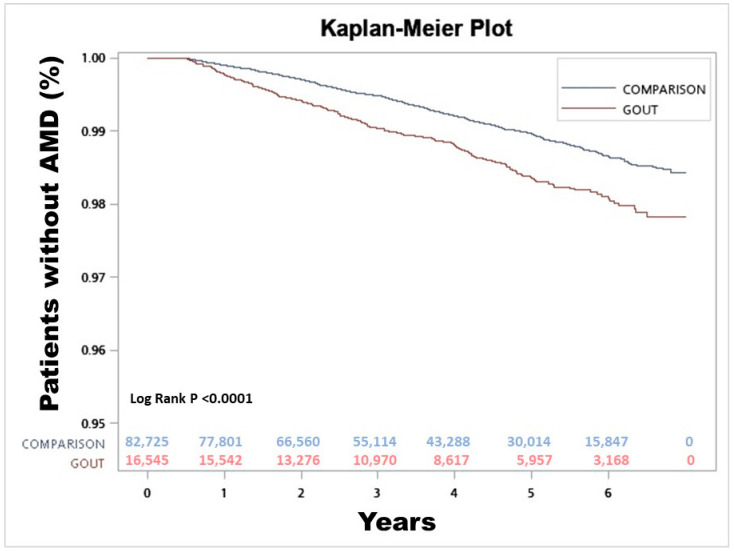
Kaplan–Meier plot comparing the development of age-related macular degeneration between gout patients and comparison group.

In this study, 209 (1.26%) patients in the gout cases group (N = 16,545) and 678 (0.82%) patients in the control group (N = 82,725) went on to develop AMD. The crude hazard ratio, determined using logistical regression with a 95% confidence interval, for gout patients to develop AMD was 1.55 (1.35, 1.78). The adjusted hazard ratio, calculated using the Cox proportional regression model, was found to be 1.20 (1.03, 1.4) (Table 2 and Table 3). The adjusted factors included age, sex, and comorbidities. Kaplan–Meier survival analysis was conducted to examine the cumulative incidence rates of AMD in the two cohorts, and a log-rank test was used to investigate the differences between the survival curves. The results were found to be significantly different, with a *p*-value of 0.0169 (Figure 1).

The crude hazard ratio of hypertension, coronary artery disease, diabetes mellitus, liver cirrhosis, glaucoma, hyperlipidemia, and migraine were also statistically significant (*p*-value < 0.05), but not uveitis. (Table 2). The results were similar for the adjusted hazard ratios (Table 3), except for coronary heart disease, which was no longer identified as a risk for developing AMD after adjustment manifest. More detailed presentations of crude and adjusted hazard ratios are provided in Table 2 and Table 3.

After matching, hypertension was found to be associated with an increased risk for both crude and adjusted hazard ratios (HR), at 1.90 (1.66, 2.17) and 1.63 (1.4, 1.89), respectively. The crude and adjusted HRs for coronary artery disease comorbidity were 1.50 (1.22, 1.83) and 1.14 (0.92, 1.42), respectively; interestingly, the HR showed no statistically significant difference after adjustment. The crude and adjusted HRs of diabetes mellitus comorbidity were 1.61 (1.36, 1.92) and 1.38 (1.15, 1.66), respectively. For liver cirrhosis comorbidity, the crude and adjusted HRs were 1.77 (1.42, 2.21) and 1.54 (1.34, 2.48), respectively. The crude and adjusted HRs of glaucoma comorbidity were 2.22 (1.66, 2.97) and 1.82 (1.34, 2.48), respectively. The crude and adjusted HRs of hyperlipidemia comorbidity were 1.75 (1.38, 2.22) and 1.45 (1.12, 1.87), respectively. The crude and adjusted HRs of uveitis comorbidity were 1.0 (0.42, 2.4) and 0.87 (0.33, 2.25), respectively. Finally, the crude and adjusted HRs of migraine comorbidity were 3.22 (1.88, 5.51) and 2.5. (1.43, 4.43), respectively.

## 4. Discussion

AMD is a disease that affects adults, particularly the elderly, and has an impact on quality of life. A recent report suggested some risk factors such as demographic and environmental risk factors as well as phenotypic risk factors were associated with the progression of AMD. [4] Investigating the etiology of AMD is essential for disease prevention and for reducing its disease burden worldwide. The results of this study show that gout is a risk factor for the development of AMD. Notably, the evidence indicates an association between these conditions; it does not prove causality. The adjusted hazard ratio of gout for AMD was found to be 1.20, meaning that a diagnosis of gout could cause a 20% increase in hazard for developing AMD in the future; the hazard ratio for Gout to develop AMD is medium. As discussed earlier, Singh et al.’s study in 2018 [12] found the adjusted hazard ratio to be 1.39, which translates to a 39% increase in hazard. Another earlier study by Klein et al. [6] investigating geographic AMD specifically found this value to be 3.48. The results of this study are therefore quite similar to that of Singh et al.’s study. It should be noted, however, that the target population used by Singh et al. was Medicare beneficiates, who were elderly individuals aged 65 years or older in the United States. In contrast, our study population included patients aged 50 years or older in Taiwan. The target outcome of Klein et al.’s study was based on pure geographic AMD; this differed from our inclusion criteria, which allowed both geographic and exudative AMD. Both our results and those of Klein et al. show an increased hazard ratio. In summary, our results are similar to those of Singh et al. and show that gout is associated with an increased likelihood of the future development of AMD. The difference in HRs between these studies may be attributed to the use of different ethnic groups and age inclusion criteria.

The association between gout and AMD could be mediated by inflammation, as gout is associated with an innate immune response caused by MSU crystal deposition in joints, which results in further systemic inflammation. This could also be the cause of the association between gout in obstructive coronary heart diseases and an increased risk of cardiovascular events [17]. Although the inflammation in AMD is largely mediated through complement-associated pathways, such as CFH, CFI, C2, CFB, and C3, other mechanisms have also been reported, including age-related maculopathy susceptibility (ARMS2), which acts through a matrix metalloproteinase inhibitor. The mechanisms involved in the interplay of these two diseases remain unclear, and further investigation is required.

Another possible mechanism for the association between gout and AMD is through oxidative stress, which is found to be higher in gout patients. Oxidative stress could result in the future development of AMD, of both exudative (neovascular) or atrophic (geographic) types. The association of oxidative stress with AMD is the reason that the Ophthalmology Society highlights in their age-related maculopathy eye disease study (AREDS) the importance of antioxidant supplementation, such as zinc, carotenoid, lutein, vitamin E, and vitamin C [18].

Another significant finding was that some comorbidities were associated with higher hazard ratios. Hypertension, hyperlipidemia, diabetes mellitus, liver cirrhosis, glaucoma, and migraine all showed increased HRs of greater than 1. The only exceptions to this were the crude and adjusted hazard ratios for uveitis and the adjusted HR for coronary heart disease, which did not achieve statistically significant differences. It can therefore be concluded that controlling hypertension, blood lipid profiles, diabetes mellitus, liver cirrhosis, glaucoma, and migraines may contribute to a difference in AMD outcome.

Our results show that gout is associated with an increased risk of developing AMD. This association may not be familiar to endocrinologists and ophthalmologists but is critical for these specialties to recognize as they function as gatekeepers for gout patients. If gout patients receive appropriate treatments and monitor their hyperuricemia diligently, the development of AMD might be avoided, and a better quality of life preserved. Recognizing this association between gout and the development of AMD would likely improve standards of public health and interdisciplinary care.

This was a population-based study performed using Taiwan’s NHIRD. The data used was representative and was sourced from a high-quality database with low selection bias. The results of this study are therefore robust. It should be noted, however, that the NHIRD is a claims database, and the diagnostic codes are based on physicians’ recordings. There is therefore no way for the researchers to verify the authenticity of the diagnoses. Furthermore, other personal data, such as body mass index, alcohol consumption, and lifestyle factors, could not be accessed. This could produce some flaws in the results of this study.

## 5. Conclusions

The NHIRD was used to investigate the association between gout and the development of AMD. The association was found to be significant, with a hazard ratio of 1.20. In terms of hypertension, hyperlipidemia, liver cirrhosis, diabetes mellites, glaucoma, and migraine, an increased hazard was shown. It is therefore imperative to effectively treat and monitor gout to prevent future AMD and to control comorbidities such as managing hypertension, blood lipid profiles, diabetes mellitus, liver cirrhosis, glaucoma, and migraines more vigorously. Hopefully, this would help contain possible future AMD development.

## Figures and Tables

**Table 1:** Baseline characteristics after matching with age and gender.

		Gout Cohort		Comparison Cohort		
		n = 16,545		n = 82,725		*p*-Value
Age (years), mean (std)		65.44	(10.16)	65.43	(10.17)	0.9525 ^a^
Age group, n (%)						0.9965 ^b^
	<55	3128	(18.91)	15,692	(18.97)	
	55~60	3117	(18.84)	15,502	(18.74)	
	60~65	2258	(13.65)	11,380	(13.76)	
	65~70	2391	(14.45)	11,883	(14.36)	
	70~75					
	≥75	3524	(21.30)	17,580	(21.25)	
Gender, n (%)						1.0000 ^b^
	Female	5518	(33.35)	27,590	(33.35)	
	Male	11,027	(66.65)	55,135	(66.65)	
Hypertension, n (%)		10,787	(65.2)	25,719	(31.09)	<0.0001 ^b^
Coronary Artery Disease, n (%)		2385	(14.42)	5898	(7.13)	<0.0001 ^b^
Diabetes Mellitus, n (%)		3547	(21.44)	9875	(11.94)	<0.0001 ^b^
Cirrhosis, n (%)		2166	(13.09)	5583	(6.75)	<0.0001 ^b^
Glaucoma, n (%)		627	(3.79)	2141	(2.59)	<0.0001 ^b^
Hyperlipidemia, n (%)		2576	(15.57)	3767	(4.55)	<0.0001 ^b^
Uveitis, n (%)		111	(0.67)	385	(0.47)	0.0006 ^b^
Migraine, n (%)		259	(1.57)	832	(1.01)	<0.0001 ^b^
AMD event, n (%)		209	(1.26)	678	(0.82)	<0.0001 ^b^
follow-up years, median (Q1, Q3)		4.15	(2.38, 5.65)	4.16	(2.4, 5.65)	0.4232 ^c^

^a^*t*-test; ^b^ chi-square test; ^c^ Wilcoxon rank-sum test.

**Table 2:** Hazard ratio estimated by univariable Cox regression stratified on matched sets.

		HR	95% CI	*p*-Value
Group				
	Comparison	1.00		
	Gout	1.55	(1.35, 1.78)	<0.0001
Hypertension, n (%)		1.90	(1.66, 2.17)	<0.0001
Coronary Artery Disease, n (%)		1.50	(1.22, 1.83)	<0.0001
Diabetes Mellitus, n (%)		1.61	(1.36, 1.92)	<0.0001
Cirrhosis, n (%)		1.77	(1.42, 2.21)	<0.0001
Glaucoma, n (%)		2.22	(1.66, 2.97)	<0.0001
Hyperlipidemia, n (%)		1.75	(1.38, 2.22)	<0.0001
Uveitis, n (%)		1.00	(0.42, 2.4)	1.0000
Migraine, n (%)		3.22	(1.88, 5.51)	<0.0001

**Table 3:** Hazard ratio estimated by multivariable Cox regression stratified on matched sets.

		Adjusted HR	95% CI	*p*-Value
Group				
	Comparison	1.00		
	Gout	1.20	(1.03, 1.4)	0.0169
Hypertension, n (%)		1.63	(1.4, 1.89)	<0.0001
Coronary Artery Disease, n (%)		1.14	(0.92, 1.42)	0.2285
Diabetes Mellitus, n (%)		1.38	(1.15, 1.66)	0.0006
Cirrhosis, n (%)		1.54	(1.23, 1.92)	0.0001
Glaucoma, n (%)		1.82	(1.34, 2.48)	0.0001
Hyperlipidemia, n (%)		1.45	(1.12, 1.87)	0.0044
Uveitis, n (%)		0.87	(0.33, 2.25)	0.7683
Migraine, n (%)		2.51	(1.43, 4.43)	0.0014

## Data Availability

Data from the National Health Insurance Research Database, now managed by the Health and Welfare Data Science Center (HWDC), can be obtained by interested researchers through a formal application process addressed to the HWDC, Department of Statistics, Ministry of Health and Welfare, Taiwan (https://dep.mohw.gov.tw/DOS/lp-2506-113.html, accessed on 1 May 2022).
